# Antibiograms and risk factors of *Salmonella* isolates from laying hens and eggs in Jimma Town, South Western Ethiopia

**DOI:** 10.1186/s13104-019-4516-5

**Published:** 2019-08-01

**Authors:** Diriba Taddese, Tadele Tolosa, Benti Deresa, Matios lakow, Abebe Olani, Eshetu Shumi

**Affiliations:** 1 Jimma University College of Agriculture and Veterinary Medicine, Jimma, Ethiopia; 2 National Animal Health Diagnostic and Investigation Center, Sebeta, Ethiopia

**Keywords:** Antibiogram, Egg, Chicken, Salmonella

## Abstract

**Objectives:**

*Salmonella* is the most important causes of foodborne illness especially from poultry and poultry products. So the aim of this study was to carryout phenotypic characterization, antimicrobials susceptibility pattern and risk factors of *Salmonella* isolates from farms and markets eggs, cloacae swabs of chickens and stool of egg collectors. A cross-sectional study was conducted from January 2018 to September 2018. Samples were, processed; *Salmonella* was isolated, phenotypically identified by OmniLog and antimicrobials susceptibility were carried out.

**Result:**

Over all; 11 (2.65%) of *Salmonella enterica* were phenotypically characterized out of 415 samples from farms egg content (n = 83), farms eggshell (n = 83), cloacae (n = 83), market eggshell (n = 83) and market egg contents (n = 83) with 2.4%, 0%, 2.4%, 4.8% and 3.6% prevalence, respectively. Out of isolates, 8 (72.72%) displayed multidrug resistance. All isolates showed susceptibility to Gentamicin, Kanamycin and Streptomycin. Lack of separating cracked eggs, washing hand, eggs stay longer unsold, and mixing excreta with feed were associated risk factors for *Salmonella* presence (P-value < 0.05). The presence of drug resistant *Salmonella enterica* within egg/and chicken can pose serious health problem. Good hygienic practices are important to reduce risk factors of *Salmonella* contamination.

## Introduction

*Salmonella* is one of the major causes of foodborne disease outbreaks globally [1]. Outbreaks due to *Salmonella* have been associated with a wide variety of foods, like; meat, chicken and egg [2, 3].

Infections can occur via ingestion of contaminated meat, eggs, raw and milk. Contamination of these foods can occur during production, processing and distribution [4]. Eggshells and egg contents can be contaminated by this bacterium during egg formation in the hen reproductive system or from environmental including fecal contact. Several outbreaks of salmonellosis have been reported where the eggs is the source of human infection [5–7].

The World Health Organization reports that, the incidence and severity of cases of salmonellosis have increased significantly [8, 9]. Some studies reported varying level of *Salmonella* prevalence (0.2–69%) in poultry [10, 11]. Bayu and his collaborators [12] report 4.69% prevalence of *Salmonella* species from egg. There was report of 41.9% prevalence of *Salmonella* from chicken farm in Jimma town [4]. Additionally antimicrobial resistance of *Salmonella* was also reported [13].

However, an egg is an important source of food; there is no report on infection/contamination status, antimicrobial susceptibility of *Salmonella* from chicken, farm and market egg in this study area. Therefore this study was designed to carry out phenotypic characterization, antimicrobial susceptibility and risk factors of *Salmonella* isolates from chicken and eggs in Jimma town.

## Main text

### Methods

#### Study area

Study was conduct in Jimma town which is situated in south western Ethiopia. Jimma town is located at latitude of 07°41″N and longitude of 36°50″ E, and at an elevation average of 1780 m above sea level [14].

#### Study design

Cross-sectional study design was conduct from January 2018 to September 2018 on egg and cloacae swab of chicken. The number of eggs sample were estimated based on previous reports using Thrusfield formula [15].

Accordingly, 4.69% [12] expected prevalence was taken with 5% desired absolute precision and 95% confidence interval. Samples size was separately calculated for eggs sampled from markets and farms.

Calculated sample size was ≈ 69 for each. This was increased by 20% and 83 eggs were sampled from each (market and farm). From 166 total eggs, 83 eggs contents and 83 eggshells of market eggs samples and 83 eggs content and 83 eggshell of farm egg samples were analyzed separately. Similarly, 83 cloacae swab samples were collected from chicken those laid sample of egg at farm. Overall, 415 samples were tested for *Salmonella* detection.

Samples from poultry farms were collected using proportional allocation sampling method and allocated samples were collected randomly. Samples of egg from markets were randomly collected. Structured questionnaire was administered to egg collectors and egg sellers at the markets to assess factors favoring contamination of egg with *Salmonella*.

#### Sample collection and transportation

Sample of egg from farms were collected as soon as egg is laid using sterile glove. Cloacae swabs were collected according to [16] and swabs were placed in sterile tube containing 10 ml of Buffered Peptone Water (BPW). Samples of egg from markets were collected using sterile glove. Each sample was coded, packaged separately in an ice box and transported to analyze laboratory.

#### Sample processing

Sterile cotton tipped swab was soaked in BPW and external egg was rubbed. Swab was inoculated in 10 ml of BPW. Eggshell was washed and immersed in 70% alcohol. Eggs were cracked and 25 g of egg content was added into flask. 225 ml of trypticase soy broth (TSB) was added on the egg content in the same flask, mixed and incubated according to [17].

#### *Salmonella* isolation and identification

*Salmonella* isolation was performed as recommended by [18]. Briefly, 1 ml of BPW mixture of eggshells, cloacae swabs and 1 ml from incubated TSB with egg content mixture were transferred to 10 ml Selenite cysteine broth (SCB) and incubated. A loop full from incubated SCB was streaked on XLD and BGA and incubated. Plate was examined for the presence of *Salmonella* [19].

*Salmonella* suspected isolates on BGA and XLD were tested via biochemical test according to [20, 21]. Isolates producing an alkaline slant with acid butt on TSI and H_2_S production or no H_2_S production, urea hydrolysis negative and indole negative, citrate utilization positive, decarboxylate lysine positive and motile were assumed as *Salmonella* species.

*Salmonella* isolates confirmed by biochemical test were taken to Biolog OmniLog test. This was by growing *Salmonella* isolates on Biolog Universal Growth Agar. Cell suspensions was made and pipette into 96 well of Biolog Plates and incubated [22]. The incubated microplates were inserted into Biolog OmniLog reader and analyzed. Result was read from computer software [23].

#### Antibiogram of *Salmonella* isolates

Phenotypically confirmed *Salmonella* isolates were subjected to 12 antimicrobial discs by agar diffusion method [24]. Culture of isolates were compared with 0.5 McFarland turbidity standards and swabbed on Mueller–Hinton Agar [4]. Antimicrobial discs were placed on Mueller–Hinton Agar and incubated. For each antimicrobial, inhibition zone was measured.

#### Associated risk factors

Structured questionnaire was pretested and administered to interviewee (farm managers, egg collectors and egg sellers at the market) to assess potential factors favoring contamination of egg with *Salmonella* species. The structured questionnaire survey at farm was includes; number of chicken in each farm, chicken keeping system, availability of disinfection bath at the entrance of the farm, eggs collection methods, feeding methods, while farm workers washing their hand after use of toilet, use of protective cloth, cleaning of stained/dirty/eggs, entrance of other people into farm, washing egg collection material/container, separating of cracked eggs from undamaged eggs, treatment of poultry with antibiotic medication, mixing of chicken excreta with fodder and eggs. The structured questionnaire survey at market was include; maximum number of days the unsold egg stays at market, using storage/frigid for unsold egg, cleanliness of egg containers, mixing eggs bought from different farmers, and separating cracked eggs.

#### Data collection, management and analysis

Data collected from laboratory investigation and questionnaire survey were stored. In univariable logistic regression, all independent variables with P-value < 0.25 were taken to multivariable logistic regression. Independent variables with P < 0.05 in multivariable logistic regression were considered as significant.

### Result

#### Phenotypically characterized *Salmonella* isolates

Over all; 11 (2.65%) out of 415 samples; *Salmonella enterica* were phenotypically characterized from farm egg content (n = 83), farm eggshell (n = 83), cloacae swab (n = 83), market eggshell (n = 83) and market egg contents (n = 83) at a rate of 2.4%, 0%, 2.4%, 4.8% and 3.6% respectively.

#### Antibiogram of *Salmonella enterica*

The degree of resistance *Salmonella enterica* ranges from 9.09 to 90.09% was observed to five antimicrobials. Of the isolates, 8 (72.72%) were multi drug resistance. Isolates susceptible to Neomycin, Ciprofloxacin, Chloranphenicol, Trimethoprim, and Tetracycline, were observed. None of the isolates resistance to Gentamicin, Kanamycin and Streptomycin was observed (Table 1).

**Table 1:** Antimicrobial susceptibility

Antimicrobial	Disc potency (μg)	Resistant (%)	Intermediate (%)	Susceptible (%)
Ceftriaxone	30	63.63	36.36	
Gentamicin	10			100
Kanamycin	30			100
Streptomycin	10			100
Neomycin	30	9.09	81.81	9.10
Ciprofloxacin	5		9.09	90.91
Chloranphenicol	30		9.09	90.91
Sulphonamides	30	90.09	9.90	
Trimethoprim	5		54.54	46.46
Tetracycline	30		9.09	90.91
Ampicillin	10	90.09	9.09	
Oxytetracycline	30	36.36	63.63	

#### Risk factors of *Salmonella* at farm and market’s egg

Risk factors for *Salmonella* contamination at farm and at market were analyzed. Hand washing before and after use of toilet, separation of cracked eggs and excreta mix with feed are factors associated with *Salmonella* contamination (P < 0.05) at farm (Table 2).

**Table 2:** Risk factors for *Salmonella enterica* at farm

Variable	Frequency (%)	Univariable		Multivariable	
		COR (95% CI)	P-value	AOR (95% CI)	P-value
Number chicken					
63	10 (3.7)	0 (0–0.032)	0.999		
106	20 (7.4)	0 (0–058)	0.998		
328	55 (20.4)	1.754 (0.006–514)	0.846		
1150	185 (68.5)	1.00			
Chicken kept system					
Cage	185 (68.5)	3.534 (0.05–2.3)	0.702		
Open	85 (31.5)	1.00			
Fodder method					
Manual	70 (100)				
Egg collecting method					
Manual	270 (100)				
Wash hand after use of toilet		1.00			
Yes	106 (39.3)	21.4 (1.285–337)	0.033	0.066 (0.005–0.809)	0.034
No	164 (60.7)	1.00			
Disinfection bath					
Yes	137 (50.7)	0.63 (0.025–15.5)	0.775		
No	133 (49.3)	1.00			
Separating cracked eggs					
Yes	119 (44.1)	28.4 (1.28–629.4)	0.034	0.062 (0.005–0.802)	0.033
No	151 (55.9)	1.00			
Dirty eggs clean					
Yes	56 (20.7)	9.57 (0.025–15.5)	0.162	0.164 (0.021–1.276)	0.084
No	214 (79.3)	1.00			
Wear special wear					
Yes	79 (29.3)	0.75 (0.030–18.7)	0.861		
No	191 (70.7)	1.00			
Other people enter farm					
Yes	139 (51.5)	0.2 (0.05–10.32)	0.451		
No	131 (48.5)	1.00			
Excreta mix with feed					
Yes	225 (83.3)	0.1 (0.003–3.1)	0.190	19.87 (2.234–176.79)	0.007
No	45 (16.7)	1.00			
Washing egg container					
Yes	159 (58.9)	0.5 (0.032–8.2)	0.635		
No	111 (41.1)	1.00			
Excreta mix with the eggs					
No	134 (49.6)	0.23 (0.016–3.12)	0.266		
Yes	136 (40.4)	1.00			
Treated with medication					
Yes	24 (8.9)	20.67 (0.612–0.700)	0.092	0.082 (0.004–1.497)	0.09
No	246 (91.1)	1.00			

The rate of *Salmonella* isolate is significantly associated (P < 0.05) with duration of unsold egg stays and separation of cracked eggs from intact one (Table 3).

**Table 3:** Risk factors for *Salmonella enterica* at market

Variable	Frequency (%)	Univariable		Multivariable	
		COR (95% CI)	P-value	AOR (95% CI)	P-value
Maximum days unsold egg stays (days)					
> 5	99 (59.6)	0.02 (0.001–0.38)	0.009	50.87 (2.65–976)	0.009
3–5	40 (24.1)	0 (0.0–0.01)	0.997		
1–2	27 (16.3)	1.00			
Unsold egg store					
No	2 (1.2)	3.6 (0.0–0.08)	1.0		
Yes	16 (498.8)	1.00			
Containers clean					
Yes	56 (33.7)	5.5 (0.4–75)	0.2	0.18 (0.013–2.455)	0.198
No	110 (66.3)	1.00			
Mix eggs bought					
No	63 (38)	9.5 (0.5–17.7)	0.129	0.106 (0.006–1.92)	0.129
Yes	103 (62)	1.00			
Separate cracked eggs					
Yes	22 (13.3)	17.97 (1.17–28)	0.038	0.055 (0.004–0.85)	0.038
No	144 (86.7)	1.00			

### Discussion

In the present study, *Salmonella enterica* was phenotypically characterized using OmniLog test. In this study, the overall prevalence (2.98%) of *Salmonella enterica* corroborates with the previous report of [25] 2.25%, and [26] 3.3% prevalence’s. However, higher prevalence of 4.64% [4], 4.69 [12], 13.88%, and 41.9% [11] were reported. Differences in prevalence rates in various studies may be due to geographic and seasonal variation, animal management practices [2] and hygienic conditions [27].

In this study, out of 13 *Salmonella enteric*; one from farm egg content and one from cloacae swab was isolated from the same chicken that might indicate as the infection of gastrointestinal gut may reason for infection of reproductive organ [28]. This could be a means for transovarial transmission of this bacterium from chicken to egg.

In the present study, occurrence of 2.41% *Salmonella enterica* species from cloacae swabs in some farms may be linked to the hygienic status of poultry production [29, 30]. The prevalence of *Salmonella enterica* species from farm egg contents in the present study was in line with 2.9% prevalence report [31]. But it shows lower prevalence when compared with 3.84% [32], 4.64% [4] and 4.69 [12]. This may be due to inadequate storage conditions of egg [33]. But in this study, eggs were collected as soon as egg laid that might minimize the exposure of egg contamination [34, 35].

In this study some of *Salmonella enterica* species from shell and contents of market egg were isolated from the same egg. This suggest as both eggs part can be contaminated with *Salmonella* from the environment [34, 35].

In this study, occurrence of 3.6% and 4.82% *Salmonella enterica* from content and shell respectively, out of analyzed sample of market’s eggs, may be due to the difference in handling/hygienic status of egg at the markets [27, 36]. This finding is in line with the studies of [32, 37].

There are reports showed drug resistances of *Salmonella* [6, 16, 32, 38]. In the current study, resistance of *Salmonella enterica* to antimicrobials is concurs with previous reports [10, 39, 40]. Multi-drug resistance observed in this study is consistent with the findings of [41, 42]. This may be due to the bacteria accumulate multiple genes; each coding for resistance [43, 44].

In this study, none of *Salmonella enterica* were resistant to Gentamicin, Kanamycin and Streptomycin is in line with [45, 46] studies. Contrary to these [4, 40], was report 100% resistance of *Salmonella* to Streptomycin. Resistivity of *Salmonella enterica* can be linked to various factors including inappropriate medication and frequent use of antibiotics [31].

In this study, importance of separating cracked egg from the intact, might be due to cracked egg promotes the gross of bacteria [28]. Similarly, mixing excreta with feed influenced the prevalence of *Salmonella* contaminates in the feed [34]. Washing hand before and after use of toilet has reduced risk for egg contamination in this study may linked with keeping hygienic status of egg collectors can minimize bacterial contamination [27]. Unsold egg stays for long time has increased risk for egg contamination may be associated with lack of appropriate use of storage and transportation [47]. However, our result suggests that establishment of good hygienic practices in poultry farm and on markets eggs are essential to reduce the contamination of *salmonella*.

## Limitation

The isolates were not molecularly characterized due to lack of resource.

## Data Availability

The data sets developed and analyzed during the current study are available from the first author or from the corresponding authors upon request.

## Abbreviations

BGA: Brilliant Green Agar
BUG: Biolog Universal Growth
XLD: Xylose Lysine Deoxycholate
